# Effect of biogenic polyamines on sliding motility of mycobacteria in the presence of antibiotics

**DOI:** 10.18699/VJGB-22-56

**Published:** 2022-08

**Authors:** I.V. Tsyganov, A.G. Tkachenko

**Affiliations:** Institute of Ecology and Genetics of Microorganisms of the Ural Branch of the Russian Academy of Sciences, Perm, Russia Perm State University, Perm, Russia; Institute of Ecology and Genetics of Microorganisms of the Ural Branch of the Russian Academy of Sciences, Perm, Russia Perm State University, Perm, Russia

**Keywords:** mycobacteria, sliding motility, antibiotic susceptibility, biogenic polyamines, микобактерии, скольжение, антибиотикочувствительность, полиамины

## Abstract

Nowadays, sliding is the least investigated mode of bacterial motility. Sliding is a process of passive movement on the surface of semi-liquid mediums which was originally described for mycobacteria and other bacterial species deprived of the organelles specialized for movement. Some mycobacteria are able to colonize surfaces, including tissues of macro-organisms, using glycopeptidolipids localized in the cell envelope for this aim. This is a serious problem for effective therapy of mycobacteriosis caused by nontuberculosis mycobacteria. Furthermore, animal tissues contain biogenic polyamines, which can increase tolerance of microorganisms to stresses, including antibiotics, and modulate cell motility. Therefore, studying mutual effects of biogenic polyamines and antibiotics on the expansion of mycobacteria is important for medicine. Mycobacterial strains, including the parent Mycolicibacterium smegmatis mc2 155 and strains containing single (ΔrelMsm) or double (ΔrelMsmΔrelZ) deletions, were used as the objects of this study. The content of glycopeptidolipids was determined using thin layer chromatography. Sliding motility was assessed by measuring the area of the sliding colony. The effectiveness of antibiotics was measured by comparison of the areas of sliding colonies in the presence of comparable concentrations of antibiotics. The polyamines spermidine and spermine had different effects on the sliding of mycobacteria through an increase or decrease in the colony areas. At the same time, polyamines had neither bactericidal nor bacteriostatic effects. The polyamines contained in the medium decreased the bactericidal effects of the antibiotics streptomycin or isoniazid, but enhanced the effects of DMNP, a synthetic analogue of the natural antibiotic erogorgiaene. Rifampicin was the most effective of all antibiotics investigated here. Moreover, we found that glycopeptidolipids are, apparently, not the only regulators of mycobacterial sliding.

## Abstract (ru)

Из всех известных способов передвижения бактерий скольжение является наименее изученным. Впервые описанное для микобактерий и некоторых других бактериальных видов, скольжение представляет собой пассивный способ перемещения по поверхности полужидких питательных сред у видов, лишенных органелл движения. Несмотря на отсутствие механизмов перемещения, некоторые микобактерии способны быстро колонизировать поверхности, в том числе ткани многоклеточных организмов, за счет присутствия в составе наружного слоя их клеточной стенки гликопептидолипидов, регулирующих силу трения о поверхность при перемещении. Это представляет серьезную проблему для эффективной терапии микобактериозов, вызываемых нетуберкулезными микобактериями. Кроме того, ткани многоклеточных животных содержат биогенные полиамины, которые способны повышать устойчивость микроорганизмов к различным стрессам, в том числе к антибиотикам, и модулировать коллективное движение. Поэтому исследование совместного действия биогенных полиаминов и антибиотиков на процессы распространения микобактерий представляет большой интерес для медицины. В качестве объектов исследования использованы штаммы микобактерий, включая родительский штамм Mycolicibacterium smegmatis mc2 155, а также его производные, содержащие одинарную (ΔrelMsm) или двойную (ΔrelMsmΔrelZ) хромосомные делеции. Содержание гликопептидолипидов определяли с помощью метода тонкослойной хроматографии. Интенсивность скольжения оценивали путем измерения площади скользящей колонии. Эффективность действия антибиотиков характеризовали сравнением площадей скользящих колоний в присутствии сопоставимых концентраций антибиотиков, выраженных в значениях, кратных минимальной подавляющей концентрации. Показано, что полиамины спермидин и спермин оказывают разнонаправленные эффекты на скольжение микобактерий по поверхностям полужидких сред, соответственно повышая или сни- жая площади колоний. При этом использовали концентрации спермидина и спермина, которые сами по себе не оказывали бактерицидный или бактериостатический эффект. Однако их совместное применение с антибиотика- ми стрептомицином или изониазидом приводило к снижению антибактериального эффекта, но усиливало дей- ствие синтетического аналога природного антибиотика эрогоргиаена (DMNP). Наиболее эффективным в этих условиях был рифампицин. Более того, нами установлено, что гликопептидолипиды, по-видимому, являются не единственными регуляторами скольжения микобактерий.

## Introduction

Sliding is a passive way of spreading bacteria on semisolid plates, which was described in 1972 (Henrichsen, 1972). At the basis of this type of motility is the action of the expansive force that occurs when dividing cells press against each other. Bacteria are pushing each other and spreading on the surface of the plate in a layer of cells through releasing surfactants in environment (Hölscher, Kovács, 2017) or accumulating glycopeptidolipids (GPLs) in cell walls (Recht et al., 2000) to decrease friction on a solid surface. Sliding motility may be realized without flagella and pili. This type of motility is available for species that were previously considered immobile. For example, in 1999, it was observed that mycobacteria can spread on semisolid pales (Martínez et al., 1999). Later, a link was established between GPLs content in mycobacterial cell walls and sliding motility. Accordingly, a model was proposed under which GPLs hydrophobic tails localized in the outer layer of the cell wall are facing the environment and so are responsible for the hydrophobic surface of cells. This hydrophobic surface doesn’t interact with hydrophilic agar plate enabling sliding for bacteria. In contrast, interaction between two hydrophobic surfaces promotes attraction between cell wall and polyvinyl chloride plates and biofilm formation (Recht et al., 2000)

Therefore, GPLs are considered to be the main factors of sliding motility in mycobacteria. However, sliding motility may be also modulated by many environmental factors like extracellular signaling molecules or plate conditions. For example, extracellular ATP secreted by damaged epithelial cells is a signaling molecule that inhibits the pulling movement of Pseudomonas aeruginosa (Nolan et al., 2015), whereas the polyamines (PA) putrescine and spermidine synthesized by Escherichia coli are required to initiate the swarming (Kurihara et al., 2009).

The role of polyamines as signaling molecules is important because these polycations are present in the cells and tissues of most living organisms, as well as water and soil. Human blood, skin, and mucous membranes also contain PAs, mainly cadaverine, spermidine, and spermine, the intracellular concentration of which can reach 2–10 mM (Gugliucci, 2004). Bacteria can synthesize putrescine, cadaverine and spermidine. PA synthesizing genes were also found in the genome of mycobacteria (Zamakhayev et al., 2018), but we have previously shown that mycobacteria can’t synthesize their own polyamines (Zamakhaev, 2020). Nevertheless, they are able to transport these polycations from the external environment. Polyamines have a positive charge and so they are able to bind to negatively charged molecules inside the cells, primarily nucleic acids, and modulate replication, transcription, translation, and other cell processes. Bacteria, getting on tissues similar to semi-solid agar in their moisture, for example, mucous membranes, are able to slide through a medium rich in biogenic polyamines, which are able to get into cells and modulate intracellular processes.

Investigation of mycobacteria sliding is important because non-tuberculous mycobacteria, the cell walls of which contain GPLs, are the cause of lung and skin infections (Tran et al., 2019). Moreover, non-tuberculous lung infections (NTLIs) have received less attention, which is likely responsible for underestimation of the incidence data on TB in the United States (Strollo et al., 2015). There are no GPLs in the cell wall of Mycobacterium tuberculosis. That is why it’s currently considered to be non-sliding. However, its cell wall contains phosphatidylinositol mannosides, phenolic glycolipids, as well as lipomannan and lipoarabinomannan (Tran et al., 2019). These lipids are also capable of creating a hydrophobic environment similar to that considered by the M. smegmatis sliding model.

Previously, we have shown that a synthetic analogue of the natural diterpene erogorgiaene, DMNP, along with widely used clinical antibiotics, has antimycobac- terial activity, and its targets are the large and small alarmone synthetases RelMsm and RelZ, which are responsible for the intracellular level of alarmone guanosine tetraphosphate (p)ppGpp (Tkachenko et al., 2021). This makes the new compound effective against the formation of quiescent cells and a promising substance for the development of new antimycobacterial drugs. Therefore, DMNP has also been a subject for studying its possible effects on M. smegmatis sliding colonies. We compared the new compound with widely used antibiotics rifampicin, streptomycin, and isoniazid, and investigated the effect of biogenic polyamines. The latter substances are represented in the natural environments and so can have a cell protective effect on mycobacteria against antibiotics (Sarkar et al., 1995).

## Materials and methods

Strains and growth media. Strains Mycobacterium smegmatis mc2 155 were objects of this study. The strain without deletion was used as a control and is indicated on the graphs as ‘WT’. Mutant strains with a single deletion of the relMsm gene and the strain with double deletions of genes relMsm and relZ were constructed on the basis of the WT strain by Sidorov R., the researcher of the Laboratory of Microbial Adaptation, Institute of Ecology and Genetics of Microorganisms RAS (Tkachenko et al., 2021). Strains were stored on Petri dishes with Luria–Bertani (LB) agar medium (Sigma, USA).

Mycobacteria were grown in a test tube with 5 mL of Middlebrook 7H9 liquid medium (HIMEDIA, India) supplemented with glycerol and 25 μg/mL ampicillin (ITW Reagents, USA) and 0.05 % Tween 80 (Rosmedbio, Russia). Cultures (5 mL) grown in tubes for 24 hours in a thermo shaker (37 °C, 200 rpm) were inoculated into 30 mL flasks with a fresh medium and cultivated under the same conditions to an optical density of 2.0–2.4.

Sliding motility. Middlebrook 7H9 medium without glycerol was solidified with 0.3 % agarose (Helikon, Russia). Polyamines and antibiotics were added to 3 mL of sterile medium that was preliminary cooled to 47 °C and dispensed per plate (40-millimeters diameter). Plates were allowed to stand at room temperature for 24 hours prior to inoculation and then 0.5 μL of liquid bacterial culture with optical density 0.2 were inoculated on the surface of the medium in the center of the plate. The cell spread area in the medium surface during growth for the indicated period of time was evaluated after plate incubation at 37 °C in a humidified box.

Measuring of the area of the sliding colony. Sliding colonies grown in plates were photographed on an Olympus C-3040 ZOOM camera (Olympus, Japan). The area was measured in pixels on photos and processed by means of the free trial version of Photoshop CC 2015.5 (Adobe, USA) as compared with the real area. The real area of one pixel was determined by comparing the diameter of the Petri plate in pixels with that of the real plate measured in millimeters.

Measuring of the optical density of the colony. The method had been described in detail earlier (Tkachenko et al., 2021). Photos of the colonies were desaturated using Photoshop CC 2015.5 to determine the optical density of the colony. The colony was singled out using the ‘quick selection tool’. ‘Brightness’ was evaluated using the ‘histogram’ tool. For illumination inversion, background brightness was taken into account. Based on the obtained background brightness values, the arithmetic mean was calculated and subtracted from the colony brightness

Determination of the minimum inhibitory concentration of the antibiotic (MIC). MIC was determined by the method of serial twofold dilutions in immunological plates (Minimed, Russia). MIC was taken as the minimal concentration at which there was no visible growth of the cell culture in the well of the plate.

Isolation of GPLs and TLC. Cells were cultured for 48 hours to achieve the stationary phase. Optical density was brought to 1.5 (600 nm). The cells were then washed from the medium and incubated in 0.6 mL of chloroform/ methanol (2:1 v/v) at 56 °C for 2 hours in a water bath sonicator (ELMA, Germany). After centrifugation (12,000 rpm, 15 min), the supernatant was purified by extraction with 600 μL distilled water. The organic phase was extracted and evaporated. Lipids were dissolved in chloroform/ methanol (9:1); 10 μL were spotted on an aluminum backed silica gel 60 TLC plate (Merck, Germany), and chromatographed with 7 mL of chloroform/methanol (9:1). The TLC plate was soaked briefly in 10 % H2SO4 in ethanol and then heated to 180 °C for 90 seconds to visualize lipids.

Biofilm formation. Mycobacterial biofilms were cultured for 48 hours in plastic plates (40 millimeters) (Medpolymer, Russia). The plates contained 4.5 mL Middlebrook 7H9 medium without Tween 80. Cells were washed from Tween 80 and 500 μL were added to plates. Results were photographed

Phase-contrast microscopy. Cells on the surface of the growth medium were visualized with a phase-contrast tool FATEK 6-7 (LOMO, Russia) and microscope MICMED-6 (LOMO). Results were photographed on the camera of an MC 6.3 microscope (LOMO).

Statistical processing of results. The results were statistically processed using the Statistica 7.0 standard software package (StatSoft Inc., USA). On the graphs, the medians (4–10 experiments) are represented, the vertical segments indicate the values of the first and third quartiles. The statistical significance of differences was assessed using the Mann–Whitney test. Differences were considered significant at p ≤ 0.05.

## Results

Influence of gene activity on the sliding motility of mycobacteria

**Fig. 1.**
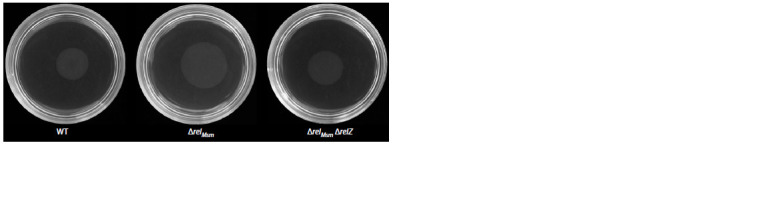
Sliding motility of mycobacteria strains.

We showed that all investigated strains were able to slide and form a monolayer of cells on agar surfaces (Fig. 1). At the same time, the control WT strain without deletions formed a colony, the area of which was smaller than that of the strain with the deletion of the relМsm gene, but differed from the strain with the double ΔrelМsmΔrelZ deletion.

**Fig. 2.**
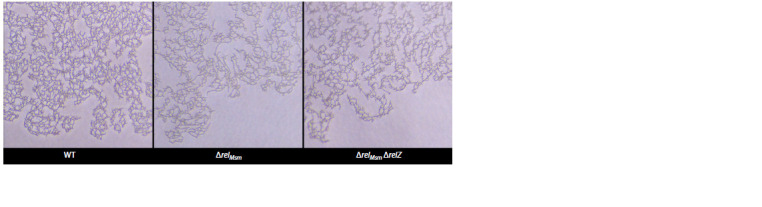
Edges of monolayers formed by sliding M. smegmatis cells of WT and deletion strains.

The study of the colony edges using phase contrast microscopy confirmed the first conclusion made on the basis of a comparison of the areas of the colonies. Cells of the strains with gene deletions were packed less densely compared to the parental strain (Fig. 2). It indicates that deletion strains are able to slide better than the parent strain.

**Fig. 3.**
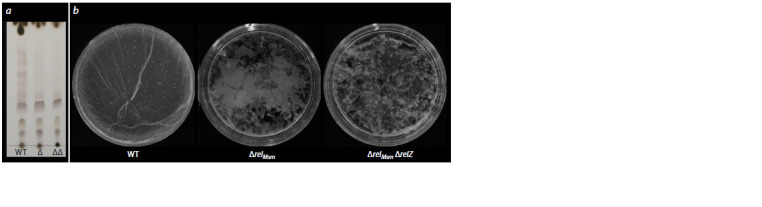
Dependency between hydrophobicity of cells and GPLs concentration in mycobacterial cell walls. a, Effect of the relМsm and relZ genes activity on the concentration of GPLs in M. smegmatis cells; b, influence of relМsm and relZ genes activity on M. smegmatis biofilm formation. Δ, Strain with a deletion of the relМsm gene; ΔΔ, strain with double deletion of the relМsm and relZ genes.

Concentration of glycopeptidolipids (GPLs) in the cell walls of mycobacteria showed that the parental strain contained the highest amount of GPLs (Fig. 3, a). The mutant strains showed the decrease in GPLs concentrations in direct proportion to the increase in the number of deletions. However, a decrease in the amount of GPLs in the cell walls of the deletion strains didn’t lead to a decrease in the area of sliding colonies (see Fig. 1). These data may indicate that either GPLs aren’t involved in a formation of hydrophobic surface for sliding or those may not be the only regulators of sliding motility in mycobacteria

Concentrations of GPLs were not only comparable with our previous results on biofilm formation in mycobacteria (Tkachenko et al., 2021), but also consistent with the information on interdependency between GPLs concentration in the cell wall and hydrophobicity of the bacterial surfaces (Recht et al., 2000). In accordance to the sliding model of mycobacteria, GPLs can form a hydrophobic cell surface, which allows a successful cell sliding through hydrophilic media. Our studies showed (see Fig. 3, b) that the cells of the control WT strain included a high concentration of GPLs in their surface structures and so were able to form biofilms which could hold on the water surface, sinking to the bottom only if their integrity was broken

In contrast, the strain with one relМsm deletion contained less GPLs and in addition to defects in biofilm formation associated with impaired activity of the alarmone synthetase gene was characterized by an ability to form the biofilm fragments that were less hydrophobic and so partially fell to the bottom of the plate. The strain with the double deletion relМsm and relZ had the least concentration of GPLs in the cell wall and the lowest hydrophobicity of the surface. Fragments of its biofilms weren’t retained on the surface and completely sank to the bottom of the plate (see Fig. 3, b). Measurement of the biomass of surface biofilms has showed no statistically significant differences between the strains (Tkachenko et al., 2021). This phase distribution of biofilm fragments was primarily dependent on the hydrophobicity of the cells.

Biogenic polyamines influence on the sliding motility of mycobacteria

**Fig. 4.**
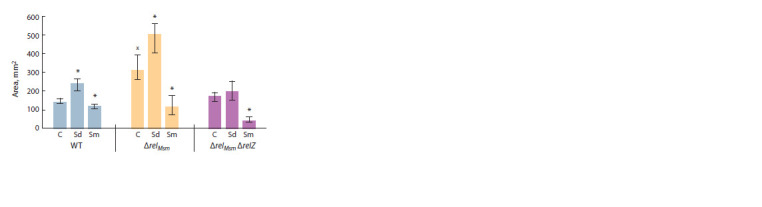
Effect of polyamines on the sliding of M. smegmatis strains. C – control without polyamine, Sd – spermidine 2 mM, Sm – spermine 2 mM. * Statistically significant difference from the control colony of the same strain grown on the medium without the addition of polyamines (Mann–Whitney test, p ≤ 0.05). х Statistically significant difference from the control colony of the strain without gene deletions (WT) grown on the medium without addition of polyamines (Mann–Whitney test, р ≤ 0.05).

Investigation of the sliding motility of the WT strain as compared to the deletion mutants showed that diameters of the sliding colonies of ΔrelМsm strain demonstrated statistically significant exceedance of those for WT control strain. However, the areas of colonies formed by the double deletion strain didn’t produce statistically significant exceedance of the areas of colonies over the control strain (Fig. 4). Biogenic polyamines spermidine and spermine, when added into the sliding medium, caused different effects. Spermidine increased the areas of colonies in the control strain, as well as in the strain with one deletion, while spermine, inversely, significantly reduced the area of sliding colonies. The polyamine effects were directly proportional to the number of deletions in the strains (see Fig. 4).

Both polyamines, spermidine and spermine, are known to have positive charges due to the presence of amino and imino groups in their molecules that are protonated at the physiological pH values (Gugliucci, 2004). However, they caused a multidirectional effect on sliding. Therefore, the effect of polyamines cannot possibly be explained by their effect on the surface charge of the cell. The decrease in the area of sliding colonies in response to addition of spermine to the cells would be interpreted as a possible bacteriostatic effect. However, as we had shown previously, the used concentrations of polyamines had no effect on the growth rate and viability of mycobacteria in a liquid medium (Tsyganov et al., 2017).

**Fig. 5.**
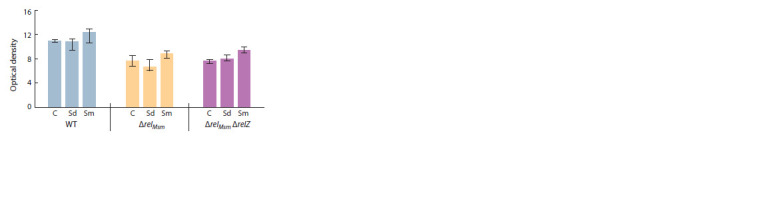
Effect of polyamines on the optical density of M. smegmatis sliding colonies. C – control without polyamine, Sd – spermidine 2 mM, Sm – spermine 2 mM.

In order to obtain more information about the effect of polyamines on the mass of sliding colonies, we tried to estimate the optical density or the number of cells in a culture. However, due to the hydrophobicity of the surface of mycobacteria grown on the medium without Tween 80, it wasn’t possible to completely separate the cells from each other, as well as to separate them from the remains of the agar medium. Therefore, an indirect assessment of the colony mass was carried out by measuring the brightness of the colonies on the shots. As a result, the optical density was standardized relative to the background values of the density of the medium surrounding the colony (Fig. 5). According to results of the measurements, it was found that the change in the areas of sliding colonies is a consequence of the polyamine effect on the sliding motility only, not on cell survival (see Fig. 5).

A statistically significant change in the areas of colonies in the presence of polyamines (see Fig. 4) didn’t change the optical density and, respectively, the mass of sliding colonies (see Fig. 5). These data support the conclusion that the polyamines spermidine and spermine had no bactericidal or bacteriostatic effects. Differences in the values of optical density between the parent strain and the strains with gene deletions are a result of concomitant changes in the growth parameters caused by changes in the genotype of the mutant M. smegmatis strains relatively to the control WT strain.

Polyamines are interfering in the sliding processes occurring in the presence of antibiotics

To investigate the effects of antibiotics on the sliding mycobacteria, we selected sublethal antibiotic concentrations, which significantly reduced the area of sliding colonies. For comparative analysis of antibiotics, all of the concentrations used were expressed as the multiplicities of the minimal inhibitory concentrations (MIC) values for the antibiotics used, which were previously determined.

**Fig. 6.**
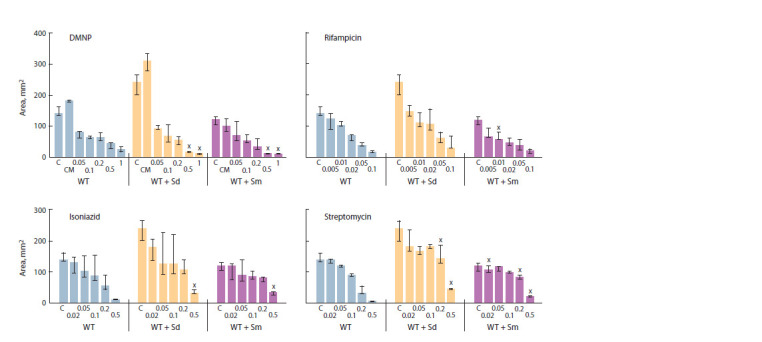
The action of polyamines and antibiotics on sliding motility of M. smegmatis control strain. Here and in the Figures 7 and 8: CM – control supplemented with 50 μL of methanol, Sd – spermidine 2 mM, Sm –spermine 2 mM. х Statistically significant difference from a similar control colony without PA (Mann–Whitney test, p ≤ 0.05)

**Fig. 7.**
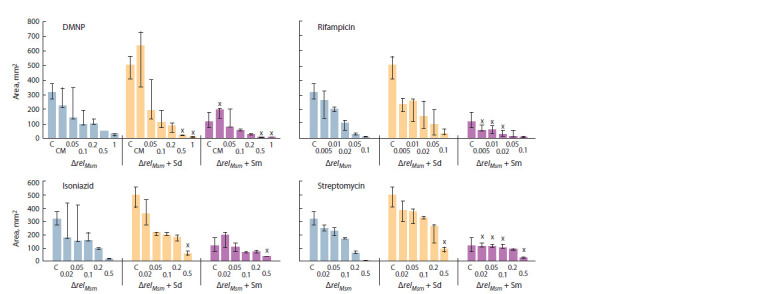
Effects of polyamines and antibiotics on sliding motility of M. smegmatis with relMsm deletion strain.

**Fig. 8.**
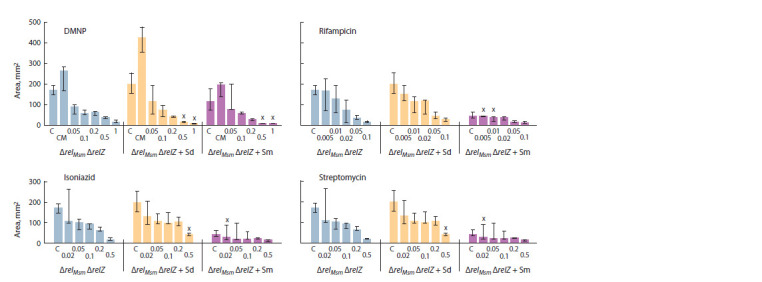
Effects of polyamines and antibiotics on sliding motility of double deletion M. smegmatis ΔrelMsm ΔrelZ.

When comparing the effectiveness of antibiotics, we have found that rifampicin most contributed to the reduction in the area of sliding colonies of all three strains of mycobacteria, while the streptomycin and isoniazid had approximately the same efficiency (Fig. 6–8). DMNP was shown to have the least antibacterial effect on growing sliding colonies, which is a consequence of its activity primarily for the stationary phase cells (Tkachenko et al., 2021).

In addition, as the DMNP was solute in methanol, we investigated the effect of methanol on the sliding motility of mycobacteria. It was shown that methanol, when added to the medium in the same volume as DMNP (50 μL), had a stimulatory effect on sliding motility as compared to the control (at the absence of methanol) (see Fig. 6–8). Spermine, as well as rifampicin and streptomycin in their minimal concentrations, had a similar inhibition activity as each of these polyamines without antibiotics. The areas of colonies were smaller than the control ones grown on the medium with antibiotic but without polyamine (see Fig. 6).

Similar results were observed for the strain with relMsm deletion (see Fig. 7). The most antibacterial effect was demonstrated by rifampicin. Spermine significantly limited the area of sliding colonies, enhancing the antibacterial effect of minimal streptomycin concentrations and rifampicin. The DMNP activity was increased in the presence of both of these polyamines and, therefore, was greater than the antibacterial effect of isoniazid and streptomycin at their maximal concentrations (see Fig. 7).

Effect of spermidine and antibiotics on the strain with a double deletion of relМsm and relZ genes was similar for that of the other two strains (see Fig. 8). At the same time, spermine reduced the sliding of mycobacteria at minimal concentrations of isoniazid, streptomycin, and rifampicin. The antibiotic DMNP with both polyamines reduced the areas of sliding colonies more strongly, but on the medium without spermine or spermidine the effectiveness of DMNP was lower than that of other antibiotics

## Discussion

The main factors of sliding are surfactants localized in bacterial cell walls or released into the external environment. Previously, it was considered that the main ones are GPLs, which are localized in the cell wall and are necessary for mycobacteria to slide on the surfaces. However, the results of our investigation on M. smegmatis strains deficient in cell wall GPLs (see Fig. 3, b) have shown that the colonies formed by the GPLs defective strains exceed the area of the colonies of the parent strain by 1.5–2 times or do not differ from them in area size. The specificity of our study is that the experimental strains with deletions did not stop synthesizing GPLs completely. Despite this, the hydrophobicity of the cell surface of the deletion strains was lower than that of the control strain, which is indirectly confirmed by the results of our studies on the nature of defects in biofilm formation in the deletion M. smegmatis strains (see Fig. 3, b). Our results suggest that GPLs are not the only regulators of mycobacterial sliding. Therefore, to determine the complete mechanism of the sliding motility, further studies are needed to investigate the role of other lipids that are the components of M. smegmatis cell wall and participate in the sliding process.

The multidirectional effect of various polyamines on the diameter of sliding colonies can’t be explained only by their influence on the electronegativity of the cell surface, since polyamines have a positive charge. At the same time, the decrease in the areas of colonies caused by spermine is also not a realization of the antibacterial effect and isn’t accompanied by a change in colony mass. This suggests that polyamines are able to modulate sliding motility by regulating intracellular processes, possibly acting as a signaling molecule, or directly through changes in cell wall composition. The determination of the sliding mechanism needs to be further investigated.

The combined effects of polyamines and antibiotics showed that rifampicin is the most effective drug against actively dividing cells in the sliding colony. DMNP showed the least activity against sliding colonies on the medium without polyamines. However, in the presence of 2 mM spermidine or spermine, the antibiotic effect was enhanced regardless of the strain of mycobacteria and exceeded that of streptomycin or isoniazid under similar conditions. Polyamines showed a protective effect at maximal concentrations of streptomycin and isoniazid.

The protective function of polyamines was previously known (Sarkar et al., 1995). Nevertheless, the sliding motility in the presence of spermine at minimal concentrations of rifampicin and streptomycin hasn’t been previously observed. The stimulatory effect of polyamines in the presence of DMNP provides this antibiotic with an advantage over a range of previously used drugs, since polyamines are widely distributed among the cells and tissues of multicellular organisms and therefore would increase the effectiveness of antibacterial drugs.

## Conclusion

In this investigation, we found that the biogenic polyamines spermidine and spermine are able to modulate the sliding motility in mycobacteria and demonstrate a multidirectional effect on this process. Spermine inhibited sliding motility at minimal concentrations of streptomycin and rifampicin. At the same time, both polyamines studied here enhanced the effect of DMNP on the diameter of colonies, making this antibiotic more effective than streptomycin and isoniazid under similar conditions. It has been shown that glycopeptidolipids, apparently, are not the only regulators of mycobacteria sliding. Therefore, the study of sliding mechanisms and the basis of polyamine effects on this process has to be further investigated

## Conflict of interest

The authors declare no conflict of interest.
